# Combining intermediate levels of the Endotoxin Activity Assay (EAA) with other biomarkers in the assessment of patients with sepsis: results of an observational study

**DOI:** 10.1186/cc11350

**Published:** 2012-05-18

**Authors:** Arino Yaguchi, Junji Yuzawa, David J Klein, Munekasu Takeda, Tomoyuki Harada

**Affiliations:** 1 Department of Critical Care and Emergency Medicine, Tokyo Women's Medical University, 8-1 Kawadacho, Shinjuku, Tokyo 1628666, Japan; 2 Department of Critical Care, and the Keenan Research Centre, Li Ka Shing Knowledge Institute, St. Michael's Hospital, University of Toronto, 30 Bond Street, Toronto, ON, M5B 1W8, Canada

## Abstract

**Introduction:**

The Endotoxin Activity Assay (EAA) is a useful test to risk stratify patients with severe sepsis and assess for Gram negative infection. However, the significance of intermediate levels of EAA (0.4-0.59) at the bedside has not been well elucidated. The purpose of this study was to interpret intermediate EAA levels in clinical practice.

**Methods:**

This retrospective observational study included all adult patients with suspected sepsis admitted to our medico-surgical intensive care unit (ICU) in whom EAA was measured from July 2008 to September 2011. Data collected included EAA, white blood cell (WBC) count and differential, C-reactive protein (CRP), procalcitonin (PCT) and bacterial cultures. Data were analyzed by comparative statistics.

**Results:**

Two hundred and ten patients were studied. Ninety two (43%) patients had culture documented gram negative infection. Patients with Gram-negative organisms in cultures had significantly higher EAA levels (0.47, IQR 0.27) than those without any Gram-negative organisms in cultures (0.34, IQR 0.22) (*p *< 0.0001). For patients with intermediate EAA levels (0.40 to 0.59), PCT levels and presence of left shift of WBC significantly differed between patients with Gram negative organisms in their blood or other cultures and those who had no organisms in any of the cultures (4.9 versus 1.7 ng/mL, *p *< 0.05; 57.9 versus 18.9%, *p *< 0.0004, respectively).

**Conclusions:**

We confirm that high levels of EAA in our cohort of patients with suspected sepsis are strongly associated with gram negative infection. In those patients with intermediate elevation in EAA levels, use of PCT and WBC differential can provide additional diagnostic value to clinicians at the bedside.

## Introduction

Endotoxin, a key component of the membrane of Gram-negative bacteria, is the most well-studied marker and mediator of sepsis. The Endotoxin Activity Assay (EAA™; Spectral Diagnostics Inc., Toronto, ON, Canada) is a rapid *in vitro *diagnostic test that is based on the reaction of neutrophils to endotoxin complexed with an anti-endotoxin antibody and that allows measurement of endotoxin activity levels in whole blood at the bedside [1-3]. The Multi-center Endotoxin Detection In Critical illness (MEDIC) trial assessed the characteristics of the EAA as a clinical test [4]. Firstly, to establish cutoff values, the assay was validated in 97 healthy controls. In this population, there was no volunteer with an EAA value of greater than 0.6, and the EAA was less than 0.4 in 93% of healthy volunteers. When conducted in 857 patients with suspected sepsis on admission to the intensive care unit (ICU), the MEDIC trial demonstrated a high value of EAA (>0.60), which was associated with an odds ratio of 3.0 for the development of severe sepsis [4]. Moreover, an EAA in the low range (<0.4) had a negative predictive value of 95.1% from risk of culture-proven Gram-negative infection, whereas an EAA in the high range (>0.6) was associated with an odds ratio of 5.3 for Gram-negative infection [4]. However, the clinical utility of EAA of between 0.4 and 0.59 was not well established by the MEDIC trial. Further clinical and diagnostic approaches are required to precisely determine the diagnosis of Gram-negative infections in patients with intermediate EAA levels. In clinical practice, we need to make an urgent diagnosis to allow the initiation of appropriate therapies such as targeted antibiotics before we obtain the results of cultures [5]. In addition, during the clinical course of patients in the ICU, the change or discontinuation of antibiotic therapy has to be quickly decided. Thus, the purpose of the present study was to elucidate the practical utility of EAA test results in the intermediate range alone and combined with other routinely and rapidly available bedside tests in ICU patients with suspected sepsis.

## Materials and methods

The study design was a single-center retrospective observational analysis of all adult patients who had sepsis and who were admitted to our tertiary academic medico-surgical ICU and in whom EAA was performed from July 2008 to September 2011. Sepsis was diagnosed by suspected infection such as by radiologic or clinical data, fever (>38.3°C) or hypothermia (<36.0°C), leukocytosis (white blood cell (WBC) count of greater than 12,000/µL) or leukopenia (WBC count of less than 4,000/µL), or plasma C-reactive protein (CRP) (>1.5 mg/dL^3^) [6]. Exclusion criteria were age of less than 18 years old and administration of steroids or other immunosuppressants [7]. Clinical and microbiological data of each patient were collected from electrical medical archives. The baseline characteristics collected were age, gender, body temperature (in degrees Celsius), WBC count and differential, CRP levels (normal is less than 0.33 mg/dL), procalcitonin (PCT) levels (normal is less than 0.5 ng/mL), and EAA (EAA™) and microbiologic culture results from any site. In keeping with our routine ICU practice, a split sample of whole blood was sent for blood culture at the time the EAA was drawn. Other cultures from sites of suspected infection were similarly sent simultaneously with EAA values. The EAA was sent (also our usual practice) on those patients with suspected sepsis of unknown origin of infection. For analysis, WBC count and differential and CRP levels were used from the time point closest to when the EAA was performed. PCT was measured on the identical blood draw as EAA. We determined that the presence of Gram-negative organisms in culture was indicative of infection if they were positive in blood, ascites, pleural effusion, or wound cultures. When culture results showed less than 1 + in sputum or less than × 10^4 ^in urine, we decided they were just colonizations.. The study protocol was approved by the Tokyo Women's Medical University institutional review board, and the need for patient consent was waived because this study is a retrospective observational analysis of patient data from medical archives.

### Statistical analysis

All values are expressed as median and interquartile ranges (IQRs). Data were analyzed by Kruskal-Wallis test, Mann-Whitney *U *test, chi-squared test, and Fisher exact probability test. A *P *value of less than 0.05 was considered to be statistically significant.

## Results

### Patient characteristics

Two hundred ten patients (141 men and 69 women; median age of 71 years, IQR of 55 to 82) were included in this study. The characteristics of patients are shown in Table 1. One hundred sixty-four patients (78%) survived to ICU discharge, and 46 (22%) patients died during their ICU stay. Twenty-seven patients had high EAA levels (≥0.6), 79 patients had intermediate levels (0.40 to 0.59), and 104 patients had low EAA levels (<0.4). For those patient groups in each EAA range, there were no significant differences in age, gender, temperature, ICU mortality, WBC count or differential, and CRP levels. PCT levels, the presence of Gram-negative organisms in any cultures, and Gram-negative organisms in blood were statistically different between high, intermediate, and low levels of EAA (4.7 versus 1.7 versus 1.0 ng/mL, *P *= 0.0003; 70.4% versus 51.3% versus 27.4%, *P *<0.0001; and 25.9% versus 10% versus 3.8%, *P *<0.0001, respectively). Although 20 patients in our cohort received polymyxin B hemoperfusion as part of their therapy, none received this treatment prior to laboratory investigations that included the EAA.

**Table 1:** Characteristics of patients

	Low EAA (<0.40)	Intermediate EAA (0.4-0.59)	High EAA (≥0.6)	*P *value
Number	104	79	27	

Age (range), years	67.5 (19-96)	71 (23-97)	76 (25-92)	0.59

Percentage of males	69.8	60	74	0.25

Body temperature (range), °C	37.0 (36.4-37.6)	37.2 (34-39.2)	37.3 (36-39.2)	0.51

White blood cells (IQR),/mm^3^	12,030 (8,720-18,150)	12,030 (7,080-17,053)	11,550 (2,522-15,888)	0.22

Stab neutrophils (IQR), percentage	9.0 (2.4-21.4)	7.5 (2.0-23.0)	10.0 (2.8-37.8)	0.45

Segmented neutrophils (IQR), percentage	78.5 (66.0-84.6)	78.0 (59.8-85.5)	71.0 (36.1- 84.8)	0.19

Left shift of neutrophils, percentage	37.7	36.3	44.4	0.77

C-reactive protein (IQR), mg/dL	11.6 (7.0-19.6)	12.2 (6.9-19.9)	13.2 (6.4-21.2)	0.06

Procalcitonin (IQR), ng/mL	1.0 (0.2-5.2)	1.7 (0.3-7.7)	4.7 (1.6-24.8)	0.003

Gram-negative organism, percentage	27.4	51.3	70.4	<0.0001

Gram-negative bacteremia, percentage	3.8	10	25.9	<0.0001

ICU mortality, percentage	17.3	22.5	37	0.13

EAA, Endotoxin Activity Assay; ICU, intensive care unit; IQR, interquartile range.

### EAA levels and Gram-negative infection

**Figure 1:**
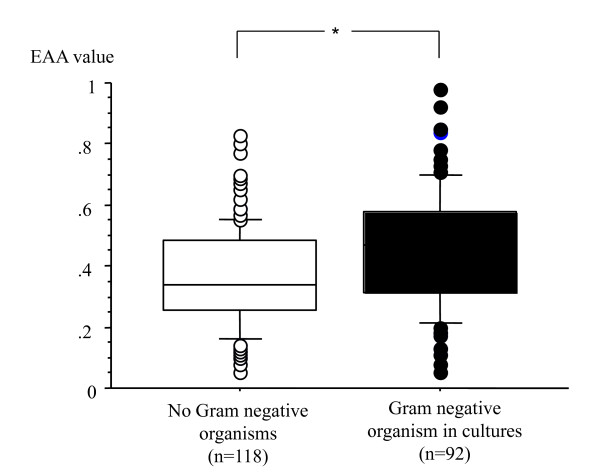
**Endotoxin Activity Assay (EAA) values in patients with Gram-negaitve infection and no infections**. Patients with no Gram-negative organisms in any culture (n = 118) (white square boxes) and with Gram-negative organisms in some cultures (n = 92) (black square boxes). The horizontal bar in each box indicates the median value, and the box indicates the interquartile ranges (IQRs). The vertical lines indicate 1.5 times the IQR. All values outside these lines are shown as black or white spots. *P < 0.0001.

**Figure 2:**
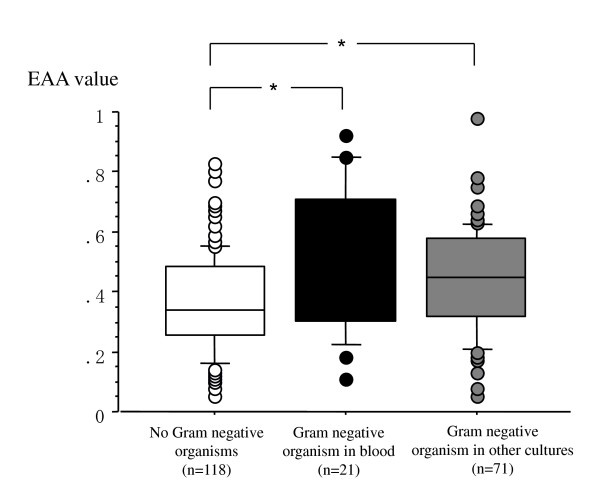
**Endotoxin Activity Assay (EAA) values and in patients with Gram-negative bacteremia and invections**. Patients with no Gram-negative organisms in any cultures (n = 118) (white square boxes), with Gram-negative organisms in some cultures without blood (n = 71) (gray square boxes), and with Gram-negative organisms in blood (n = 21) (black square boxes). The horizontal bar in each box indicates the median value, and the box indicates the interquartile range (IQR). The vertical lines indicate 1.5 times the IQR. All values outside these lines are shown as black, gray, or white spots. *P < 0.001.

EAA levels in patients with Gram-negative organisms in any culture (n = 92) and without any Gram-negative organisms in any cultures (n = 118) were significantly different (0.47, IQR 0.27 versus 0.34, IQR 0.22, *P *<0.0001) (Figure 1). There were also statistically significant differences between patients with Gram-negative organisms in blood (n = 21) and those without any Gram-negative organisms in any of the cultures (n = 118) - 0.50 (IQR 0.44 to 0.71) versus 0.34 (IQR 0.26 to 0.48), respectively, *P *= 0.0007 - and between patients with Gram-negative organisms in culture that were not bacteremic (n = 71) and those without any Gram-negative organisms in any of the cultures (n = 118) - 0.45 (IQR 0.32 to 0.58) versus 0.34 (IQR 0.26 to 0.48), respectively, *P *= 0.001 - (Figure 2).

### Organisms and intermediate EAA

Table 2 shows the distribution of bacterial organisms in cultures and associated EAA levels. Of the 113 culture examinations of patients with intermediate EAA levels, 10 blood cultures revealed Gram-negative organisms and 9 blood cultures were positive for Gram-positive organisms. In sputum, 32 cultures had Gram-negative species, 17 cultures had Gram-positive species, and 5 cultures were positive for fungus. There were 9 cultures from wound or abscess, 17 cultures from urine, and 3 cultures from ascites of Gram-negative organisms in the intermediate group.

**Table 2:** Distributions of the organism in the cultures and the levels of Endotoxin Activity Assay

Culture sample		Organism	Low EAA (<0.4)	Intermediate EAA (0.4-0.59)	High EAA (≥0.6)
Blood	Gram-negative	*Escherichia coli*	3	4	
		*Klebsiella pneumoniae*		4	4
		*Klebsiella oxytoca*			1
		*Bacteroides fragilis*	1	1	1
		*Pseudomonas aeruginosa*		1	
		*Serratia marcescens*			1
	Gram-positive	*Streptococcus pyogenes*	2	1	1
		*Staphylococcus epidermidis*	1	1	1
		*MSSA*	4	6	1
		*MRSA*	5	1	
		*Enterococcus faecalis*	1		
Suptum	Gram-negative	*Escherichia coli*	2	2	1
		*Pseudomonas aeruginosa*	3	4	4
		*Enterobacter aergenes*		2	
		*Enterobacter cloacae*	1	2	
		*Klebsiella pneumoniae*	1	9	4
		*Klebsiella oxytoca*			1
		*Acinetobacter*	2	2	1
		*Serratia marcescens*	4	3	2
		*Stenotrophomonas maltophilia*	4	8	2
		*Proteus mirabilis*	2		
		*Haemophilus influenzae*	1		
	Gram-positive	*Streptococcus pneuminiae*	6	2	
		*Staphylococcus aureus*	2	2	
		*Corynebacterium sp*.	2	2	
		*MSSA*	13	3	
		*MRSA*	9	7	4
		*Enterococcus faecalis*	3	1	2
	Fungus	*Candida albicans*	18	5	
Urine	Gram-negative	*Escherichia coli*	3	7	2
		*Pseudomonas aeruginosa*	1	5	2
		*Klebsiella pneumoniae*	4	3	3
		*Klebsiella oxytoca*	1	2	
		*Citrobacter freundii*	2		
	Gram-positive	*Streptococcus*	1		
		*Enterococcus*	3		
		*MSSA*		2	
		*MRSA*	1		
	Fungus	*Candida albicans*	3	3	
Ascites	Gram-negative	*Escherichia coli*	1	2	
		*Bacteroides fragilis*		1	
Pleural effusion	Gram-negative	*Pseudomonas aeruginosa*	1		
		*Klebsiella pneumoniae*	2		
	Gram-positive	*MRSA*	3	2	
Abscess, wound	Gram-negative	*Pseudomonas aeruginosa*	1	1	
		*Bacteroides fragilis*	2	3	
		*Klebsiella pneumoniae*	1	3	
		*Escherichia coli*	2	2	1
	Gram-positive	*MSSA*	2	1	1
		*MRSA*	3		
		*Streptococcus*	5	1	
		*Enterococcus faecalis*	2	2	

EAA, Endotoxin Activity Assay; *MRSA*, methicillin-resistant *Staphylococcus aureus*; *MSSA*, methicillin-sensitive *Staphylococcus aureus*.

### Patient characteristics in the intermediate EAA levels (0.4 to 0.59)

There was no statistically significant difference for age, gender, body temperature, WBC count, CRP levels, and EAA values between patients with Gram-negative organisms detected in cultures (n = 42) and those without any Gram-negative organisms in any cultures (n = 37) (Table 3). The percentage of stab and segmented neutrophils, left shift of neutrophils, and PCT levels were statistically different: 18% (IQR 3.5% to 32.0%) versus 3.5% (IQR 1.4% to 7.8%), *P *= 0.0006; 65.0% (IQR 48.5% to 78.5%) versus 86.0% (IQR 70.5% to 86.5%), *P *= 0.0004; 57.9% versus 18.9%, *P *= 0.0004; and 4.9 (IQR 0.4 to 13.8) versus 1.7 (IQR 0.2 to 3.0) ng/mL, *P *= 0.05, respectively.

**Table 3:** Characteristics of patients in the intermediate Endotoxin Activity Assay levels

	Gram-negative organism in cultures (n = 42)	No Gram-negative organism in any cultures (n = 37)	*P *value
Age (range), years	76 (23-97)	71.0 (32-92)	0.10
Percentage of males	69	51	0.11
Body temperature (range), °C	37.4 (34.6-39.2)	37.4 (34.0-39.0)	0.98
White blood cells (IQR),/mm^3^	11,070 (6,130-17,480)	12,410 (6,740-16,290)	0.95
Stab neutrophils (IQR), percentage	18 (3.5-32.0)	3.5 (1.4-7.8)	0.0006
Segmented neutrophils (IQR), percentage	65.0 (48.5-78.5)	83.0 (70.5-86.5)	0.0004
Left shift of neutrophils, percentage	57.9	18.9	0.0004
C-reactive protein (IQR), mg/dL	13.1 (6.0-21.4)	13.7 (6.6-19.4)	0.91
Procalcitonin (IQR), ng/mL	4.9 (0.4-13.8)	1.7 (0.2-3.0)	0.05
EAA value (IQR)	0.5 (0.45-0.53)	0.49 (0.45-0.52)	0.88

EAA, Endotoxin Activity Assay; IQR, interquartile range.

### Combining PCT levels, left shift of neutrophils, and Gram-negative organisms in patients with intermediate EAA levels (0.4 to 0.59)

Patients with EAA levels of 0.4 to 0.59 were divided into two groups according to the appearance of left shift of neutrophils or PCT values of more than 5 ng/mL or both. The presence of Gram-negative organisms was compared between the two groups (Table 4). There were 36 patients (45.6%) with left shift of neutrophils or PCT levels of more than 5 ng/mL or both and 43 patients (54.4%) without left shift of neutrophils or PCT levels of less than 5 ng/mL or both. Of the 36 patients, 25 patients were positive for Gram-negative organisms in any culture; of the 43 patients without left shift of neutrophils or PCT levels of less than 5 ng/mL or both, 26 patients were negative for Gram-negative organisms in any culture. A statistically significant difference was shown for the presence of Gram-negative organisms between the groups (*P *= 0.0008). The positive predictive value (PPV) was 59.5%, whereas the negative predictive value (NPV) was 70.2%. In the low level of EAA, the PPV and the NPV were 29.1% and 72.7%, respectively. In the high level of EAA, the PPV and the NPV were 82.4% and 50.0%, respectively.

**Table 4:** Left shift of neutrophils and procalcitonin levels and Gram-negative organism infection in the intermediate Endotoxin Activity Assay level

	Gram-negative organism in cultures (n = 42)	No Gram-negative organism in any cultures (n = 37)
Left shift (+) or PCT (≥ 5.0 ng/mL) or both	25	11
Left shift (−) or PCT (<5.0 ng/mL) or both	17	26

*P *= 0.008. PCT, procalcitonin.

## Discussion

This study confirms that EAA levels are significantly increased in patients with culture-proven Gram-negative infection. In the low-EAA group, Gram-negative organisms were found in sputum, urine, ascites, pleural effusion, and wounds but rarely in blood cultures. This result is consistent with that of previously reported studies [3,4]. A particular difference of our work is that the EAA measurement and other laboratory data as well as blood cultures were done on identical blood samples. Therefore, the present results could be more reliable than those of previous studies, which showed differences between the results of the EAA and incidence of documented Gram-negative infections [3,8]. Similar to previous investigations, our study is limited in that the study patients had the decision whether to perform an EAA made on the basis of clinical bedside assessment of suspicion of sepsis rather than studying all ICU patients. Several patients had already received empiric antibiotics in their clinical course and this may confound the results given that the potential impact of different antibiotics on different organisms in terms of changing lipopolysaccharide levels is not well understood. In clinical practice, we usually have to determine whether our patient has sepsis and whether antibiotics should be initiated, changed, or stopped in empirical cases without any documented microbiology. Therefore, EAA may be expected to have additional utility as one of the tools for diagnosis of sepsis. Recently, several studies have shown the utility of EAA in the evaluation of the severity, prognosis, and indications of therapy for sepsis [3,9-14]. It has been generally established that high EAA levels (≥0.6) can help rule in sepsis due to Gram-negative infections and that patients with low EAA levels (<0.4) have a low risk of sepsis due to Gram-negative infection. The issue of the interpretation of EAA results in the intermediate range remains. Clinically, these patients may have sepsis due to Gram-negative infection or other sources of infection with translocation of endotoxin from the gastrointestinal tract [15]. The patients may also have endotoxemia as a result of translocation of endotoxin from another disease process. Risk of endotoxemia has been shown in patients with trauma, major burns, transplantion, pancreatitis, or other conditions that may predispose to gut translocation [15-17]. Our study showed that PCT levels and the percentage of stab neutrophils were increased in patients with documented Gram-negative organisms in comparison with those without any documented Gram-negative organisms. Therefore, we combined PCT levels and left shift of neutrophils with EAA to evaluate for diagnosis of Gram-negative sepsis in the patient group with intermediate levels of EAA. Plasma PCT level alone is limited in its diagnostic ability for sepsis at the bedside, as are older tests like WBC differential [6]. There are a few studies regarding the correlation between EAA and PCT; one study shows that there are concomitant elevations of EAA levels and PCT in patients with sepsis [18], whereas another study shows no correlation between EAA and PCT [19]. The former study showed that EAA levels (>0.60) and PCT (>2.0 ng/mL) had a positive agreement with a clinical diagnosis of severe sepsis [18]. In those patients with confirmed Gram-negative infection, our study demonstrates a strong correlation between EAA in the high range and PCT. Furthermore, we show that in patients who have intermediate levels of EAA (0.4 to 0.59), the addition of PCT levels and presence of a left shift of neutrophils can be useful to diagnose sepsis due to Gram-negative infection.

## Conclusions

The EAA is a rapid and simple test that is used at the bedside to evaluate patients with suspected sepsis for endotoxemia in clinical practice. High EAA levels (≥0.6) can be used to diagnose sepsis due to Gram-negative infections, and low EAA levels (<0.4) can be used to rule out Gram-negative infections. Combining plasma PCT levels and left shift of neutrophils can aid clinicians at the bedside in interpreting the meaning of an EAA in the intermediate range. Further research is necessary to prospectively validate this model in the ICU setting.

## Key messages

• High Endotoxin Activity Assay (EAA) levels (≥0.6) can be used to help diagnose sepsis due to Gram-negative infections.

• Low EAA levels (<0.4) can be used to rule out Gram-negative infections.

• Procalcitonin levels and presence of shift to the left of white blood cells (WBCs) show differences between Gram-negative infection and no Gram-negative infection in intermediate EAA levels.

• Combining procalcitonin and differential of WBCs can help to diagnosis sepsis in intermediate EAA levels.

## Abbreviations

CRP: C-reactive protein; EAA: Endotoxin Activity Assay; ICU: intensive care unit; IQR: interquartile range; MEDIC: Multi-center Endotoxin Detection In Critical illness; NPV: negative predictive value; PCT: procalcitonin; PPV: positive predictive value; WBC: white blood cell.

## Competing interests

DJK has served as a consultant to Spectral Diagnostics Inc., manufacturer of the EAA. All other authors declare that they have no competing interests.

## Authors' contributions

AY designed, carried out, and performed the statistical analysis and drafted the manuscript. JY conceived of the study, performed the assay, and acquired data. DJK analyzed and interpreted data and helped to draft the manuscript. MT and TH participated in study design and acquired data. All authors read and approved the final manuscript.

## Authors' information

AY, MT, and TH hold MD and PhD degrees. JY is a medical technologist. DJK holds MD and MBA degrees.
